# Extracellular vesicle-mediated delivery of miR-101 inhibits lung metastasis in osteosarcoma

**DOI:** 10.7150/thno.33482

**Published:** 2020-01-01

**Authors:** Kailiang Zhang, Chuan Dong, Ming Chen, Tongtao Yang, Xin Wang, Yongheng Gao, Lijuan Wang, Yanhua Wen, Guanyin Chen, Xinli Wang, Xiuchun Yu, Yinglong Zhang, Pingshan Wang, Mingfu Shang, Kang Han, Yong Zhou

**Affiliations:** 1 Department of Orthopedic Surgery, Orthopedic Oncology Institute of Chinese PLA, Tangdu Hospital, Air Force Medical University, Xi'an 710032, Shaanxi, China; 2 Rehabilitation Center of Lintong Sanatorium of PLA, Xi'an 710600, Shaanxi, China; 3 Department of Respiratory, Tangdu Hospital, Air Force Medical University, Xi'an 710032, Shaanxi, China; 4 Department of Dermatology, the First Affiliated Hospital of Medical College of Xi'an Jiaotong University, Xi'an, 710061, Shaanxi, China; 5 Department of Orthopedics, the 960th Hospital of the PLA Joint Logistics Support Force, Jinan 250000, Shandong, China; 6 Department of Orthopedics, the Fourth Medical Center of Chinese PLA General Hospital, Beijing, 100048, China

**Keywords:** extracellular vesicle, mesenchymal stromal cell, miR-101, osteosarcoma

## Abstract

**Rationale:** Extracellular vesicles (EVs) have emerged as novel mediators of cell-to-cell communication that are capable of the stable transfer of therapeutic microRNAs (miRNAs), and thus, EVs hold immense promise as a miRNA delivery system for cancer therapy. Additionally, as miRNA-containing EVs are secreted into circulation, miRNAs contained within plasma EVs may represent ideal biomarkers for diseases. The objective of this study was to characterize a potential tumor suppressor miRNA, miR-101, and explore the potential of miR-101 delivery via EVs for *in vivo* therapy of metastatic osteosarcoma as well as the potential value of plasma EV-packaged miR-101 (EV-miR-101) level for predicting osteosarcoma metastasis.

*Methods:* The relationship of miR-101 expression and osteosarcoma progression was investigated in osteosarcoma specimens by in situ hybridization (ISH), and the potential inhibitory effect of miR-101 was further investigated using *in vivo* models. Using prediction software analysis, the mechanism of action of miR-101 in osteosarcoma was explored using quantitative reverse transcription polymerase chain reaction (qRT-PCR), western blotting and dual-luciferase assay. Adipose tissue-derived mesenchymal stromal cells (AD-MSCs) were transduced with lentiviral particles to obtain miR-101-enriched EVs. A Transwell assay and lung metastasis models of osteosarcoma were used to observe the effect of miR-101-enriched EVs on osteosarcoma invasiveness and metastasis. Detection of plasma EV-miR-101 levels was carried out in osteosarcoma patients and healthy controls by qRT-PCR.

**Results:** miR-101 expression was markedly lower in metastatic osteosarcoma specimens compared to non-metastatic specimens. Significantly fewer metastatic lung nodules were formed by Saos-2 cells overexpressing miR-101 and SOSP-9607 cells overexpressing miR-101 injected into mice. With increased miR-101 expression, B cell lymphoma 6 (BCL6) mRNA and protein expression levels were reduced, and miR-101 was found to exert its effects by directly targeting BCL6. AD-MSCs were successfully engineered to secrete miR-101-enriched EVs. Once taken up by osteosarcoma cells, these EVs showed suppressive effects on cell invasion and migration *in vitro*, and systemic administration of these EVs effectively suppressed metastasis *in vivo* with no significant side effects. Finally, the EV-miR-101 level was lower in osteosarcoma patients than in healthy controls and even lower in osteosarcoma patients with metastasis than in those without metastasis.

**Conclusion:** Our data support the function of miR-101 as a tumor suppressor in osteosarcoma via downregulation of BCL6. AD-MSC derived miR-101-enriched EVs represent a potential innovative therapy for metastatic osteosarcoma. EV-miR-101 also represents a promising circulating biomarker of osteosarcoma metastasis.

## Introduction

Osteosarcoma is among the most common primary malignant tumors of bone and predominantly occurs in children and adolescents 1. It carries a high risk of distant metastasis, especially lung metastasis, and the 5-year survival rate is less than 30% for osteosarcoma patients with lung metastasis 2-4. At present, there are no effective treatment strategies or predictive biomarkers for osteosarcoma metastasis. To achieve a better prognosis, it is vital that we discover the molecular mechanisms responsible for osteosarcoma metastasis. Such knowledge would allow the identification of reliable biomarkers for predicting osteosarcoma metastasis and support the development of effective therapeutic agents.

MicroRNAs (miRNAs) are a class of non-coding small RNA molecules, and in the context of cancer, both tumor-suppressing miRNAs and oncogenic miRNAs have been discovered 5. As a tumor-suppressing miRNA, miR-101 is frequently downregulated in several types of cancer and invariably plays a suppressive role in cancer progression 6-8. In our previous study, miR-101 was shown to inhibit the invasion and migration of osteosarcoma cells *in vitro*, partly via regulation of the enhancer of zeste homolog 2 (EZH2) 9. For miR-101 to exert such inhibitory effects against osteosarcoma cells *in vivo* and prevent metastasis, targeted delivery of miR-101 to osteosarcoma cells is necessary, which requires a safe and effective vehicle.

Extracellular vesicles (EVs), known as exosomes, are widely accepted generic terms for particles with a lipid bilayer that are naturally secreted from cells, and examples include endosome-originating “exosomes” and plasma membrane-derived “ectsomes” (microparticles/microvesicles) 10. The potential use of EVs as miRNA delivery vehicles has received increasing attention, as EVs naturally contain miRNAs that can be transferred through EV uptake and release to mediate the function of recipient cells both locally and distantly 11-15. Moreover, EVs can be loaded with a specific miRNA by packaging the miRNA into parent cells through viral and non-viral methods 16, 17.

The parent cell must be selected carefully, because the characteristics and contents of EVs are reflective of the cell of origin 16. Mesenchymal stromal cells (MSCs) have been shown to possess the capacity to evade the host immune response by affecting the function of immune cells in the context of both adaptive and innate immunity 18-21 and to have a natural tropism for tumors and their metastases 22, 23. In addition, among the various cell types known to secrete EVs, MSCs are the most prolific producer 24. These characteristics of MSCs have prompted interest in the use of MSC-derived EVs as tumor-specific vehicles for miRNA delivery. MSCs have been successfully engineered to secrete miRNA-enriched EVs, which were shown to have therapeutic effects against some tumors both *in vitro* and *in vivo* 25-29. Specifically, delivery of miR-143-enriched EVs from engineered MSCs resulted in significant inhibition of osteosarcoma cell migration *in vitro* 30.

In addition to the therapeutic effects of EV-packaged miRNAs (EV-miRNAs), researchers are also pursuing their potential as diagnostic tools based on evidence that EV-miRNAs are functionally related to cancer progression, metastasis, and aggressive tumor phenotypes, in combination with their stability in bodily fluids 31. Several EV-miRNAs have shown promise for the diagnosis of cancers 32-34.

We hypothesized that EV-mediated transfer of miR-101 from engineered MSCs can inhibit the invasiveness and metastatic ability of osteosarcoma cells and that EV-packaged miR-101 (EV-miR-101) in plasma may serve as a useful circulating biomarker for predicting osteosarcoma metastasis. In the present study, we began testing these hypotheses by investigating the biological function and mechanism of action of miR-101 in osteosarcoma metastasis, the therapeutic efficacy of EV-mediated delivery of miR-101 *in vitro* and *in vivo*, and finally the potential value of plasma EV-miR-101 detection for the diagnosis of osteosarcoma metastasis.

## Results

### Downregulation of miR-101 is correlated with metastatic progression and poor prognosis in osteosarcoma patients

**Figure 1:**
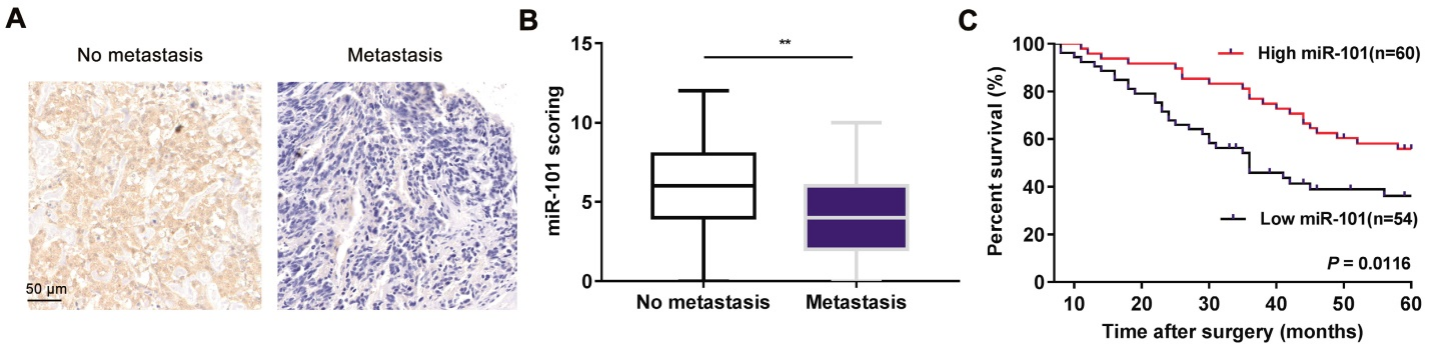
** Decreased miR-101 expression is associated with osteosarcoma metastasis and predicts a poor prognosis. (A)** Representative images of miR-101 ISH in osteosarcoma specimens from non-metastatic and metastatic osteosarcoma specimens (magnification, 200×). **(B)** Statistical comparison of differences in miR-101 expression between non-metastatic and metastatic osteosarcoma specimens. Student's *t*-test. Error bars show minimum to maximum values. **(C)** Relationship between miR-101 expression and overall survival time of osteosarcoma patients. Log-rank test. ^**^P<0.01.

To explore the role of miR-101 in osteosarcoma metastasis, in situ hybridization (ISH) was conducted to evaluate miR-101 expression. Our data showed that miR-101 expression was significantly lower in metastatic osteosarcoma specimens than in non-metastatic specimens (Fig. 1A-B). The 114 osteosarcoma patients were divided into high and low miR-101 expression groups based on the ISH scores for miR-101 expression in osteosarcoma specimens. As shown in Table 1, the low miR-101 expression group showed a higher metastatic rate and more advanced clinical stage compared with the high miR-101 expression group. In addition, the low miR-101 expression group also had a shorter overall survival time than the high miR-101 expression group (Fig. 1C). Collectively, decreased miR-101 expression was predictive of a poor prognosis and may be implicated in the stimulation of osteosarcoma metastasis.

### miR-101 exhibits a suppressive effect against osteosarcoma metastasis in *in vivo* models

**Figure 2:**
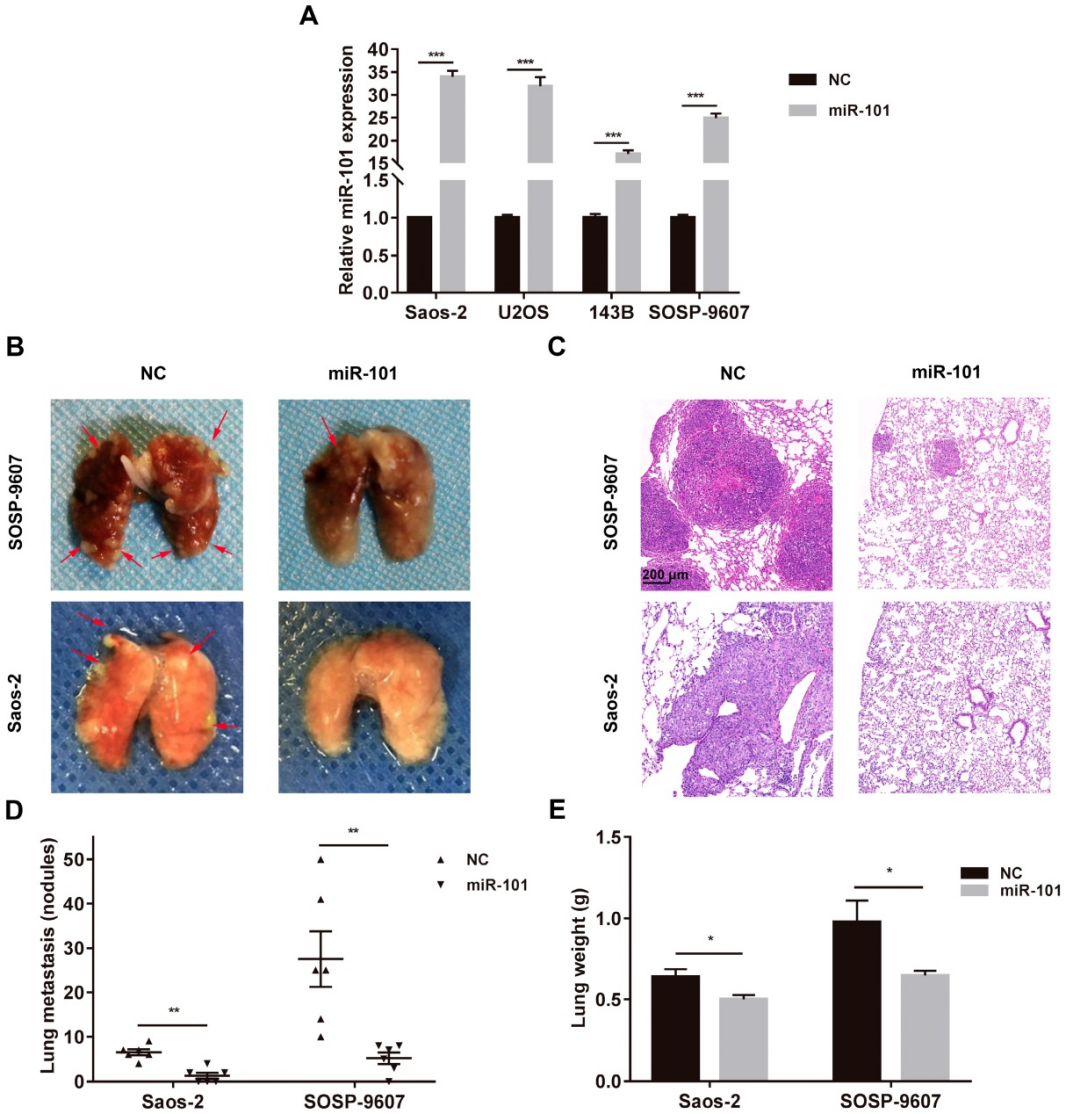
** miR-101 inhibits osteosarcoma metastasis in *in vivo* models. NC, negative control. (A)** Confirmation of upregulated miR-101 expression following lentiviral vector-mediated transduction of osteosarcoma cell lines. Student's *t*-test. **(B)** Representative images of lung metastases, with arrowheads indicating metastatic nodules. The male Balb/c nude mice (6 weeks old) were divided into two groups (n= 6 per group), and then 3×10^6^ of the indicated cells were administered via tail vein injection. All mice were sacrificed 7 weeks after cell injection. **(C)** Representative hematoxylin and eosin (H&E) staining of metastatic lung nodules (magnification, 100×). **(D)** Statistical analysis of the number of metastatic lung nodules in each model. Mann-Whitney test. **(E)** Lung weight in nude mice injected with the different osteosarcoma cells. Student's *t*-test. ^*^P<0.05; ^**^P<0.01; ^***^P<0.001.

To investigate whether miR-101 could inhibit lung metastasis *in vivo*, osteosarcoma cell lines (SOSP-9607 and Saos-2) stably expressing miR-101 were constructed through lentiviral vector-mediated transduction, and the transduction efficiency was measured by quantitative reverse transcription polymerase chain reaction (qRT-PCR; Fig. 2A). To establish *in vivo* models of osteosarcoma metastasis, SOSP-9607 cells expressing miR-101 (SOSP-9607-101 cells), SOSP-9607 cells transduced with the negative control (NC) virus particles (SOSP-9607-NC cells), Saos-2 cells expressing miR-101 (Saos-2-101 cells), and Saos-2 cells transduced with the NC virus particles (Saos-2-NC cells) were injected into the tail vein of nude mice (n=6 per group) to produce lung metastasis. Seven weeks later, the mice were sacrificed, and the results showed significantly fewer metastatic lung nodules and a significantly lower lung weight in the animals injected with SOSP-9607-101 or Saos-2-101 cells compared to their counterparts injected with the NC-transduced cell lines (Fig. 2B-E). In addition, to exclude the effect of miR-101 on cell proliferation, which may affect the formation of lung metastases *in vivo*, the proliferation of SOSP-9607-101 and Saos-2-101 cells after lentiviral vector-mediated transduction was evaluated using the CCK-8 assay and compared to that of the NC cells. As shown in Fig. S1A-B, miR-101 expression had little effect on the proliferation of these osteosarcoma cells, which was consistent with the findings of our previous work 9.

### BCl6 is a direct target of miR-101 in osteosarcoma cells

**Figure 3:**
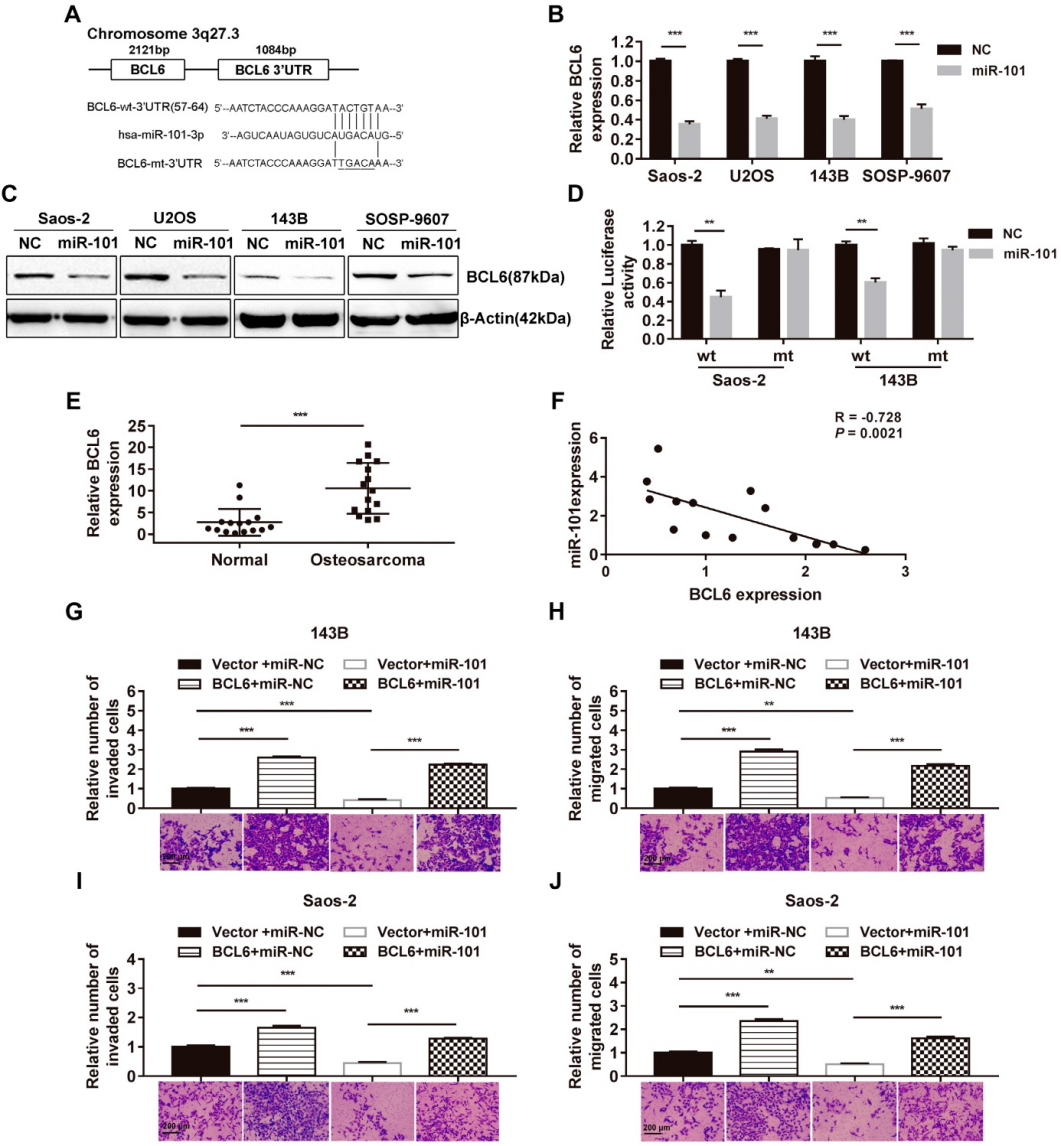
** miR-101 exerts inhibitory effects on osteosarcoma cells by targeting BCL6. NC, negative control. (A)** The complementary sequences between miR-101 and the BCL6 3'UTR as well as the mutations are shown. **(B-C)** Effect of miR-101 on BCL6 mRNA and protein expression as determined using qRT-PCR and western blotting in osteosarcoma cells. Student's *t*-test. **(D)** Effect of miR-101 expression on luciferase activity of Saos-2 and 143B cells after transfection with the wild-type or mutant BCL6 3'UTR reporter gene. Student's *t*-test. **(E)** Evaluation of BCL6 mRNA expression in osteosarcoma tissues versus adjacent normal tissues using qRT-PCR. Student's *t*-test. **(F)** Assessment of the correlation between miR-101 and BCL6 mRNA expression in osteosarcoma tissues by Pearson's correlation coefficient analysis. **(G-J)** Effect of restoration of BCL6 expression on Saos-2 and 143B cell invasion and migration observed via Transwell assay. One-way analysis of variance (ANOVA) and Tukey's multiple comparisons test. ^*^P<0.05; ^**^P<0.01; ^***^P<0.001.

Given the sequence homology between miR-101 and the 3' untranslated region (3'UTR) of BCL6, the effect of elevated miR-101 expression on BCL6 mRNA and protein expression was also assessed, and reductions in BCL6 expression at both the mRNA and protein levels were found in SOSP-9607-101 cells, U2OS cells expressing miR-101 (U2OS-101), Saos-2-101 cells and 143B cells expressing miR-101 (143B-101) cells, relative to their NC counterparts (Fig. 3A-C). In addition, a dual-luciferase reporter assay was performed to test whether BCL6 is a direct target of miR-101. The results showed that elevated miR-101 expression significantly suppressed luciferase activity of the wild-type BCL6 3'UTR reporter gene in 143B and Saos-2 cells, but not that of the mutant reporter gene in the respective cell lines (Fig. 3D). In addition, qRT-PCR analysis of BCL6 expression in 15 pairs of matched osteosarcoma tissues and adjacent normal tissues showed increased BCL6 mRNA levels in osteosarcoma tissues compared to adjacent normal tissues (Fig. 3E). The correlation between miR-101 and BCL6 expression was also investigated in the 15 osteosarcoma specimens, and the results showed that miR-101 expression was inversely correlated with BCL6 mRNA expression (Fig. 3F). In contrast to the function of miR-101, BCL6 significantly enhanced osteosarcoma cell invasion and migration, and ectopic expression of BCL6 rescued the suppression of osteosarcoma cell invasion and migration induced by miR-101 (Figs. 3G-J and Fig. S2). Taken together, these data demonstrate that miR-101 inhibits cell invasion and migration by directly targeting BCL6.

### miR-101 can be successfully packaged into adipose tissue-derived mesenchymal stromal cell (AD-MSC)-derived EVs

Confirmation of the purity of the isolated AD-MSCs (Fig. S3A) was performed by flow cytometric analysis of the expression of positive and negative MSC markers, including CD31, CD34, CD44, CD45, CD90 and CD105 (Fig. S3B).

After 48 h of lentiviral vector-mediated transduction at a multiplicity of infection (MOI) of 40, green fluorescent protein (GFP) expression was observed in AD-MSCs (Fig. S3C). The flow cytometric analysis of the expression of MSC markers was repeated, and no significant differences were observed between the engineered AD-MSCs and native AD-MSCs.

To further investigate the multi-lineage differentiation potential of the engineered AD-MSCs, differentiation of the cells into adipocytes, chondrocytes and osteocytes was induced, and then Oil red O, Alcian blue and Alizarin red S staining, respectively, were performed to identify the differentiated cells. As shown in Fig. S3D-F, engineered AD-MSCs retained the same multi-lineage differentiation potential as the native AD-MSCs.

**Figure 4:**
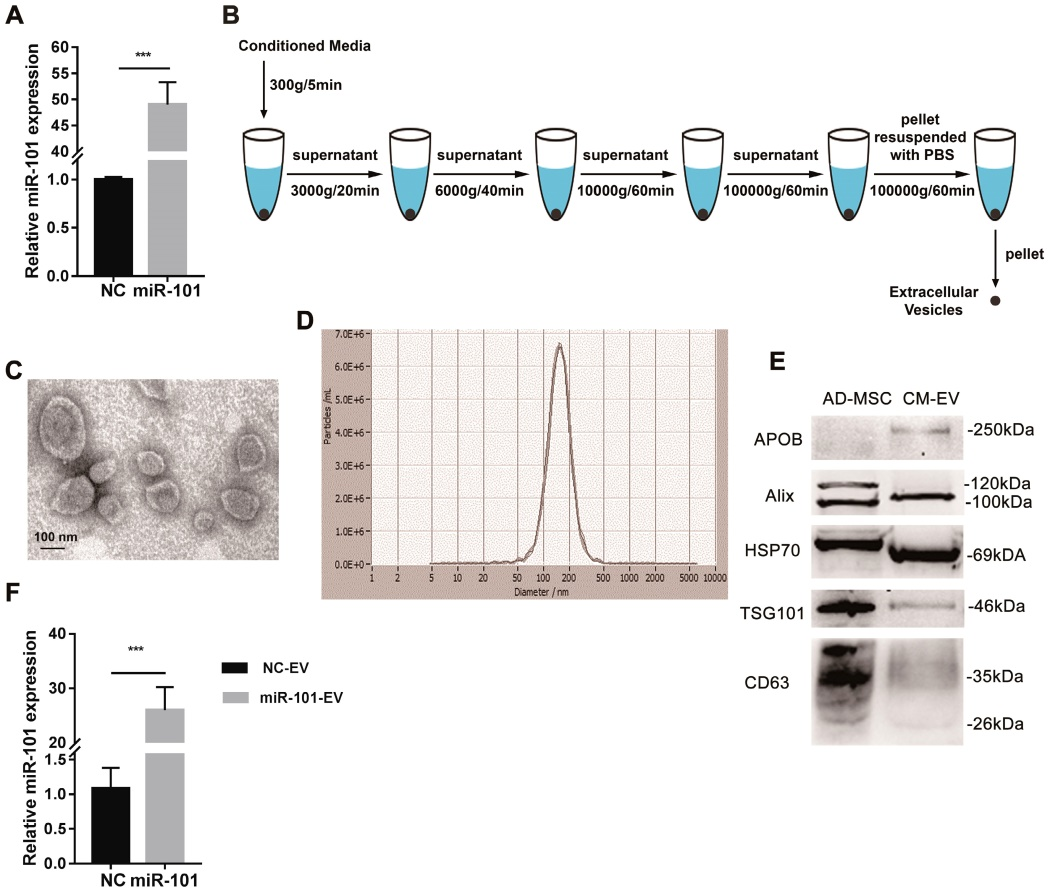
** AD-MSCs were successfully engineered to secrete miR-101-enriched EVs. NC, negative control; AD-MSC, adipose tissue-derived mesenchymal stromal cell; CM-EV, EVs derived from conditioned media of AD-MSCs; NC-EV, EVs derived from AD-MSCs-NC; miR-101-EV, EVs derived from AD-MSCs-101. (A)** Transduction efficiency of miR-101 was measured by qRT-PCR. Student's *t*-test. **(B)** Steps of differential centrifugation. **(C)** Representative TEM image of AD-MSC-derived EVs. **(D)** NTA results for AD-MSC-derived EVs (n=3). **(E)** Expression of surface markers of EVs by western blotting. Equal amounts (2 μg) of protein were loaded in each lane, and AD-MSC lysate was used as a control for EV characterization. **(F)** Relative amounts of miR-101 in AD-MSC-101-EVs and AD-MSC-NC-EVs. Student's *t*-test. ^***^P<0.001.

The expression of miR-101 was proven to be increased in AD-MSCs expressing miR-101 (AD-MSCs-101) compared to AD-MSCs transduced with NC virus particles (AD-MSCs-NC) by qRT-PCR (Fig. 4A). Differential centrifugation was applied to obtain EVs from conditioned media (Fig. 4B), and the AD-MSC-derived EVs were examined by transmission electron microscopy (TEM; Fig. 4C) and nanoparticle tracking analysis (NTA; Fig. 4D). Positive (i.e., CD63, tumor susceptibility gene 101 [TSG101], heat shock protein 70 [HSP-70] and Alix) and negative (i.e., apolipoprotein B [APOB]) protein markers of EVs were also detected by western blotting (Fig. 4E). The amount of miR-101 contained within AD-MSC-101-derived EVs (AD-MSC-101-EVs) was significantly greater than that in AD-MSC-NC-derived EVs (AD-MSC-NC-EVs; Fig. 4F).

### miR-101-enriched EVs from engineered AD-MSCs inhibit osteosarcoma cell invasion and migration *in vitro*

**Figure 5:**
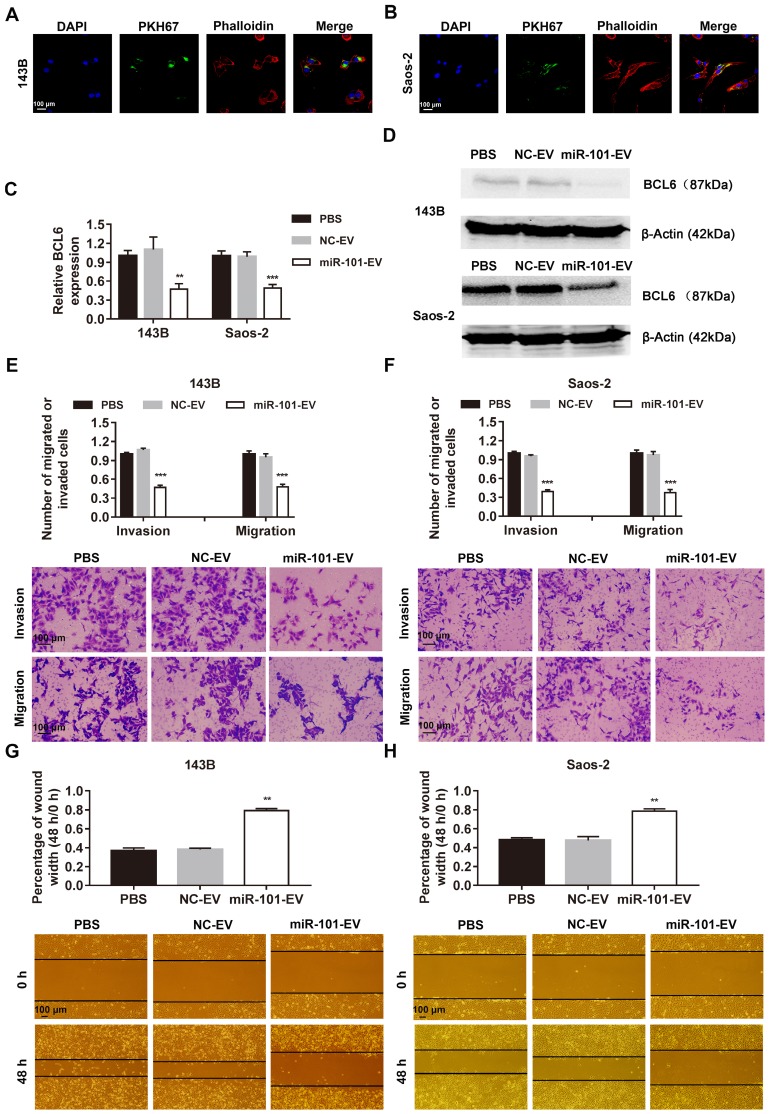
** AD-MSC-101-EVs suppressed the invasion and migration abilities of osteosarcoma cells. NC-EV, EVs derived from AD-MSCs-NC; miR-101-EV, EVs derived from AD-MSCs-101. (A-B)** Uptake of miR-101-EVs by 143B and Saos-2 cells as observed using confocal microscopy (magnification, 400×). **(C-D)** Effect of AD-MSC-101-EVs on BCL6 mRNA and protein expression as determined by qRT-PCR and western blotting, respectively, in 143B and Saos-2 cells. One-way ANOVA and Tukey's multiple comparisons test. **(E-F)** Effect of AD-MSC-101-EVs on invasion and migration of 143B and Saos-2 cells in a Transwell assay (magnification, 200×). One-way ANOVA and Tukey's multiple comparisons test. (G-H) Effect of AD-MSC-101-EVs on the migration of 143B and Saos-2 cells as observed in a wound healing assay (magnification, 40×). One-way ANOVA and Tukey's multiple comparisons test. ^**^P<0.01 and ^***^P<0.001 compared with PBS or NC-EV treatment.

As shown in Fig. 5A-B, AD-MSC-101-EVs were efficiently taken up by 143B and Saos-2 cells, as evidenced by increased miR-101 levels in 143B and Saos-2 cells (Fig. S4). This uptake subsequently led to decreased BCL6 mRNA and protein expression (Fig. 5C-D). Osteosarcoma cells treated with AD-MSC-101-EVs displayed significantly reduced invasion and migration abilities compared to cells treated with AD-MSC-NC-EVs or an equal volume of phosphate-buffered saline (PBS; Figs. 5E-F and Fig. S5). The results of the wound healing assay for 143B and Saos-2 cell migration were consistent with those of the Transwell assay (Fig. 5G-H).

### miR-101-enriched EVs from engineered AD-MSCs inhibit osteosarcoma metastasis *in vivo*

**Figure 6:**
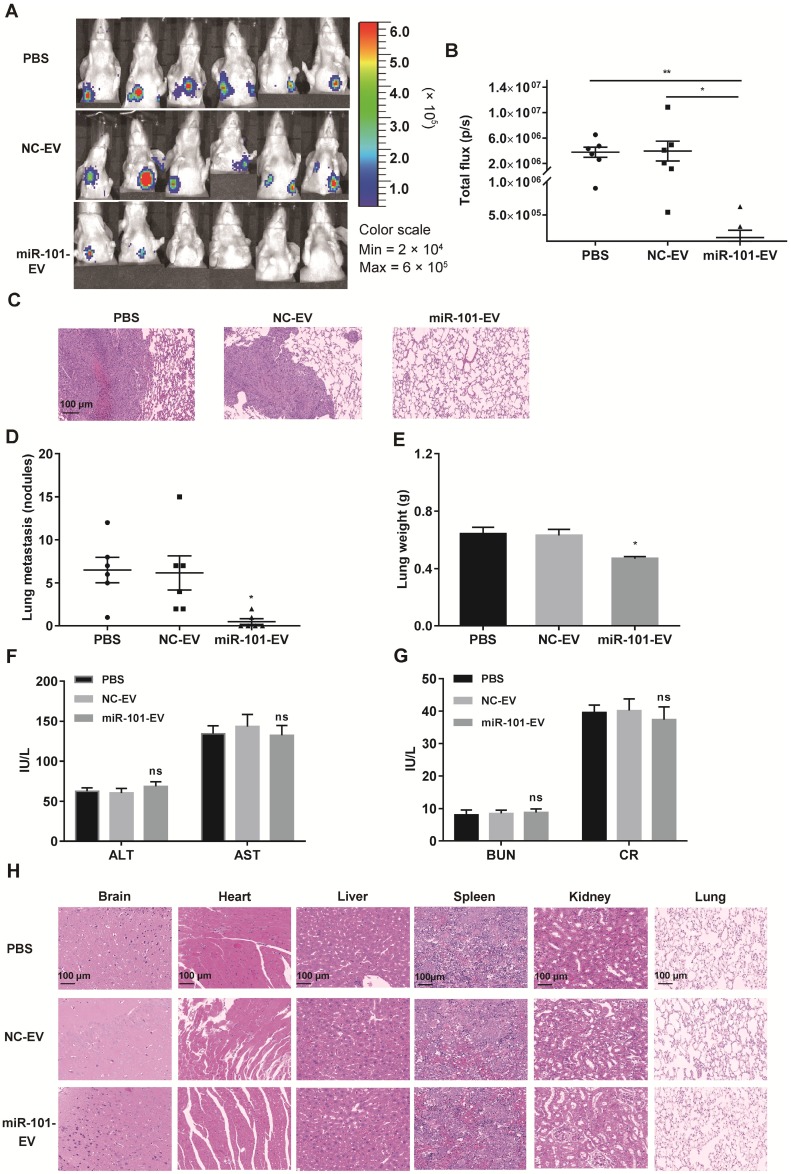
** AD-MSC-101-EVs suppressed lung metastasis of osteosarcoma in nude mice models. NC-EV, EVs derived from AD-MSCs-NC; miR-101-EV, EVs derived from AD-MSCs-101; ALT, alanine aminotransferase; AST, aspartate transaminase; BUN, blood urea nitrogen; CR, creatinine. (A)** Lung metastases in the three groups was evaluated by an *in vivo* imaging system (IVIS) at 7 weeks after tumor cell injection (n=6 per group). **(B)** Quantification of bioluminescence imaging. p/s: photons per second. Kruskal-Wallis test and Dunn's multiple comparisons test. **(C)** H&E staining of metastatic lung nodules (magnification, 100×). **(D)** Numbers of metastatic lung nodules. Kruskal-Wallis test and Dunn's multiple comparisons test. **(E)** Lung weight for the nude mice. One-way ANOVA and Tukey's multiple comparisons test. **(F-G)** Detection of ALT, AST, BUN, and CR levels among the three groups. One-way ANOVA and Tukey's multiple comparisons test. (H) H&E staining of brain, heart, liver, spleen, kidney and lung (magnification, 200×). ns, not significant (*P* >0.05). ^*^ P <0.05 in comparison with PBS or NC-EV treatment.

To verify whether miR-101-enriched EVs from engineered AD-MSCs have clinical potential for the treatment of osteosarcoma metastasis *in vivo*, 143B cells stably expressing luciferase (143B-luci) were injected into the proximal tibia of nude mice to form tumors. Beginning 2 weeks later, AD-MSC-101-EVs, AD-MSC-NC-EVs or an equal volume of PBS were injected into the tail vein of mice two times a week for 1 month. In contrast to the mice that received AD-MSC-NC-EVs or PBS, which all developed lung metastases, only two of six mice (2/6) that received AD-MSC-101-EVs developed lung metastases within 7 weeks after tumor cell injection (Figs. 6A-B and Fig. S6A-D). Upon sacrifice of the mice, the number of metastatic lung nodules and lung weight were significantly less in the mice that received AD-MSC-101-EVs than in those that received AD-MSC-NC-EVs or PBS treatment (Fig. 6C-E).

To evaluate the *in vivo* safety of EV treatment, another 15 nude mice were randomly divided into three groups (n=5 per group), which also received AD-MSC-101-EVs, AD-MSC-NC-EVs or an equal volume of PBS. At 1 week after the final injection treatment, all mice were sacrificed. Testing for serological markers showed no significant difference among the three groups (Fig. F-G), indicating the EV treatment had no harmful effects on hepatic-renal function. H&E staining of major organs was also performed and showed no obvious toxicity in mice that received AD-MSC-101-EVs, AD-MSC-NC-EVs or PBS treatment (Fig. 6H).

### Plasma EV-miR-101 shows potential as a diagnostic biomarker for osteosarcoma

**Figure 7:**
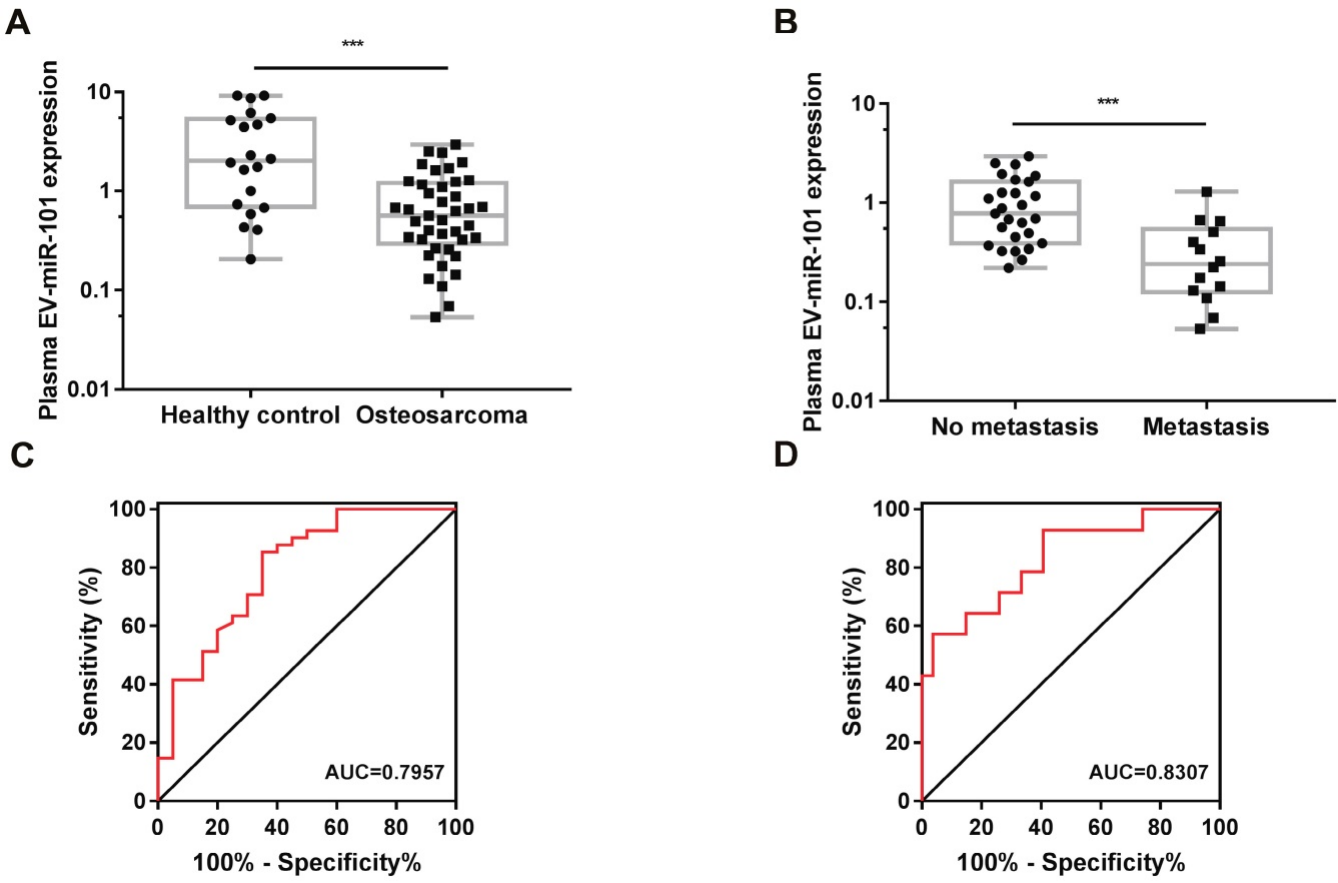
** The plasma EV-miR-101 level was reduced in osteosarcoma patients compared with healthy controls and even lower in osteosarcoma patients with metastasis than in those without metastasis. (A)** A lower EV-miR-101 level was observed in osteosarcoma patients compared to healthy controls. Mann-Whitney test. Error bars show minimum to maximum values. **(B)** A lower EV-miR-101 level was observed in osteosarcoma patients with metastasis compared to patients without metastasis. Mann-Whitney test. Error bars show minimum to maximum values. **(C)** ROC curve analysis for the use of plasma EV-miR-101 for differentiating osteosarcoma patients from healthy controls. **(D)** ROC curve analysis for the use of plasma EV-miR-101 for differentiating osteosarcoma patients with metastasis from those without metastasis. ^***^P<0.001.

The results of next-generation sequencing (NGS) for a plasma sample from an osteosarcoma patient (compared with a plasma sample from one healthy control) demonstrated the existence of miR-101 in plasma EVs, as well as a decreased level of miR-101 in the plasma EVs of the osteosarcoma patient compared with the healthy control (Table 2). To further verify the diagnostic potential of EV-miR-101, a total of 61 plasma specimens (Table S1) from osteosarcoma patients (n=41) (no metastasis at diagnosis) and healthy controls (n=20) were examined. Plasma EVs were obtained by differential centrifugation and examined by TEM, NTA and western blotting (Fig. S7A-C). Lower EV-miR-101 expression was found in osteosarcoma patients relative to healthy controls (Fig. 7A). Moreover, compared to osteosarcoma patients without metastasis (n=27), patients with metastasis (n=14) showed even lower miR-101 levels (Fig. 7B).

Receiver operating characteristic (ROC) curve analysis was then performed to evaluate the potential usefulness of plasma EV-miR-101 as a noninvasive biomarker for the diagnosis of osteosarcoma. The results showed that the EV-miR-101 level was effective for discriminating patients with osteosarcoma from healthy controls (area under the ROC curve [AUC]=0.7957, 95% confidence interval [CI]=0.6715-0.9199, *P*=0.0002; Fig. 7C). In addition, the plasma EV-miR-101 level could distinguish osteosarcoma patients with metastasis from those without metastasis (AUC=0.8307, 95% CI=0.6959-0.9654, *P*=0.0006; Fig. 7D).

In addition, the level of plasma EV-packaged let-7i-5p (EV-let-7i-5p) was also detected and showed no significant difference between healthy controls and osteosarcoma patients (Fig. S8A). Moreover, the plasma EV-let-7i-5p level did not differ significantly between osteosarcoma patients without metastasis and those with metastasis (Fig. S8B). These results suggest that EV-let-7i-5p can be used as an endogenous control for future studies of osteosarcoma markers.

## Discussion

Our previous research revealed a tumor suppressing role of miR-101 in relation to osteosarcoma invasiveness and metastasis *in vitro* 9. In the current study, miR-101 expression was further detected in osteosarcoma specimens and found to be downregulated in metastatic osteosarcoma specimens compared with non-metastatic specimens. The formation of fewer metastatic pulmonary nodules by Saos-2 and SOSP-9607 osteosarcoma cells overexpressing miR-101 in xenograft models, established by tail vein injection, was observed, mainly demonstrating that miR-101 can suppress homing of circulating tumor cells to the lung. Together with our previously published work, the results of the present study clearly show the metastasis-suppressing properties of miR-101 in osteosarcoma, suggesting the therapeutic potential of miR-101 against osteosarcoma metastasis.

Based on analyses using two programs (Targetscan Version 7.2 and G: profiler), the oncogene BCL6 was chosen as a potential target of miR-101. The transcription factor BCL6 was originally identified as an oncogene in B-cell lymphomas and then found to be involved in a wide range of hematologic and solid tumors 40, 41. Our results showed that miR-101 expression was inversely correlated with BCL6 expression in osteosarcoma tissue samples. Moreover, *in vitro* experiments using osteosarcoma cells stably expressing miR-101 showed decreased BCL6 mRNA and protein expression, and BCL6 was demonstrated to be a novel target of miR-101 by a dual-luciferase reporter assay. The inhibitory effect of miR-101 on osteosarcoma cell invasion and migration was rescued by restoration of BCL6 expression. Our data confirm the existence of a miR-101/BCL6 axis for the first time, as well as the oncogenic role of BCL6 in osteosarcoma. Prior research revealed that due to the synergic effect of BCL6 and EZH2 in lymphoma, targeting BCL6 and EZH2 together leads to a great therapeutic effect 42. Considering our present results with our previous data^9^, we have confirmed miR-101 exerts its function partly through regulation of BCL6 and EZH2 expression. The potential synergistic function between oncogene BCL6 and EZH2 in osteosarcoma requires further investigation.

Collectively, our findings definitively demonstrate the suppressive effect of miR-101 on osteosarcoma metastasis, which sparks interest in EV-mediated delivery of miR-101 *in vivo* to inhibit metastasis in osteosarcoma patients. Here, AD-MSCs were successfully engineered to produce miR-101-enriched EVs for miR-101 delivery. Subsequent *in vitro* experiments showed reduced invasive and migratory abilities of osteosarcoma cells after incubation with AD-MSC-miR-101-EVs for 48 h. The incubation time with EVs was chosen based on the time required for changes in protein expression to appear after uptake of AD-MSC-miR-101-EVs by cells. Multiple incubation methods with different incubation times have been reported for Transwell assays in the literature 43-46. To determine the optimal conditions for achieving the desired level of inhibition, it may be necessary to do a time course analysis of the effect of EVs on osteosarcoma cell invasion and migration under specific experimental conditions.

One of the most important findings in this study is the potential effectiveness of delivering AD-MSC-101-EVs for the treatment of osteosarcoma *in vivo*, which is consistent with the findings of previous studies showing effective treatment of multiple malignancies using engineered MSC-derived EV-miRNAs *in vivo* 26, 27, 45, 47. It has become clear that a relationship between protein quantification and the number of EVs does not exist, and therefore, we conducted EV quantification using NTA, which is considered the best method currently available 41. Based on previous studies, repeated intravenous injections were performed to ensure the best chance for a therapeutic effect 26, 47, 48. Due to an innate ability of MSCs to home to tumors and their metastases, MSC-derived EVs may reflect the tumor-homing capacity of the parent cell 22, 23, thereby obtaining the desired results. Our findings were in accordance with the results of a study showing that systemically administered MSC-derived EVs did not cause an evident immune reaction in several organs of animals 49. Because MSCs have the capacity to evade the host immune response 18-20, our results suggest that EVs from AD-MSCs may also reflect that capacity.

EV-miRNAs have been used as a novel diagnostic tool for a series of malignancies 31, 50. Our clinical data also support the role of plasma EV-miR-101 as a circulating biomarker for osteosarcoma. It has been reported that over 50% of EVs in serum are released by platelets during the process of clot formation 51, and thus, plasma was chosen, instead of serum, for the isolation of EVs in our study. To check for possible platelet contamination, routine blood testing was conducted for the plasma samples (obtained just before the step of ultracentrifugation to pellet the EVs), and no platelets were detected (data not shown).

Recently, serum miR-101 expression has been proposed as a promising diagnostic marker of osteosarcoma 52. It is necessary to compare the effectiveness of miRNAs and EV-miRNAs as circulating biomarkers of cancers. It is well established that EVs can protect miRNAs from RNase degradation and facilitate stable, long-term storage of miRNAs 53, 54. Importantly, EVs have some tissue-specific membrane markers, which can assist in detecting tumor origin 55. Thus, the use of EV-miRNAs as circulating biomarkers for cancer detection has shown great promise.

In summary, we have demonstrated that miR-101 is a definitive suppressor of osteosarcoma invasiveness and metastasis, and these effects are mediated, at least in part, through regulation of BCL6. EVs derived from engineered AD-MSCs show great potential as an *in vivo* delivery vehicle of miR-101 for this disease. The plasma EV-miR-101 level also holds exciting potential as a new biomarker of osteosarcoma metastasis, although our study was limited by a small sample size due to the rarity of these samples. Further research is needed to determine the clinical value of the plasma EV-miR-101 level in additional, larger patient cohorts. The findings of this study provide insight into the potential use of EV-miR-101 within novel diagnostic and therapeutic strategies for osteosarcoma patients with metastasis.

## Methods

### Human osteosarcoma specimens and plasma specimens

The experimental protocol was approved by the Institutional Review Board of Tangdu Hospital, and informed consent was obtained from all participants. In total, 114 formalin-fixed, paraffin-embedded human osteosarcoma tissue specimens, 41 plasma specimens, and 15 paired osteosarcoma and normal adjacent tissue specimens were obtained from patients diagnosed with osteosarcoma based on histopathological evaluation at Tangdu Hospital. None of the patients received any therapy before biopsy or blood collection. Plasma specimens were also obtained from 20 healthy individuals.

### Cell culture

The human osteosarcoma cell line SOSP-9607 was established by our laboratory 35. U2OS and Saos-2 cells were maintained in our laboratory and cultured in Dulbecco's Modified Eagle's Medium (DMEM; Hyclone), while 143B (American Type Culture Collection) and SOSP-9607 cells were cultured in Minimal Essential Media (MEM; Gibco) and RPMI-1640 media (Hyclone), respectively. All culture media contained 10% fetal bovine serum (FBS; Sijiqing, China) and 5 µg/ml Cellmaxin plus (GenDEPOT, USA). All cells were incubated at 37 °C with 5% CO_2_.

### Construction of stable cell lines

The lentiviral particles (4×10^8^ TU/ml; lentiviral vector: pGV309- hU6-MCS-Ubiquitin-EGFP-IRES-puromycin) encoding miR-101 or the empty vector as the NC were purchased from Genechem (Shanghai, China). Cells were seeded into 6-well plates, and after 24 h, the cells were transduced with lentiviral particles in complete medium supplemented with 40 μl HitransG P (Genechem) for 12 h according to the supplier's instructions. The U2OS, Saos-2 and 143B cells were transduced at a MOI of 50, whereas The MOIs for SOSP-9607 cells and AD-MSCs were 100 and 40, respectively. All cells were cultured in complete media supplemented with 2 μg/ml puromycin (Solarbio, China) for selection. GFP expression was evaluated at 48 h after lentiviral vector-mediated transduction under a fluorescence microscope (Olympus, Japan).

For *in vivo* bioluminescent imaging, 143B cells were transduced under the same conditions (MOI 40, 2 μg/ml puromycin) as described above to express a luciferase gene. The lentiviral particles (5×10^8^ TU/ml; lentiviral vector: pGV260-Ubiquitin-MCS-Luc_firefly-IRES-puromycin) expressing firefly luciferase were purchased from Genechem.

### RNA extraction and qRT-PCR

RNA was extracted from EVs using TRIzol LS reagent (Invitrogen), while cellular and tissue RNA was isolated with TRIzol reagent (Sigma-Aldrich). Isopropanol was added to the RNA extracted from EVs with glycogen (Thermo), and the mixture was stored at -20 ℃ overnight. Glyceraldehyde-3-phosphate dehydrogenase (GAPDH), U6, Cel-mir-39 and let-7i-5p were used as controls. The corresponding primers were: GAPDH: forward: 5'-AATCCCATCACCATCTTCCA-3'; reverse: 5'-TGGACTCCACGACGTACTCA-3'; and BCL6: forward: 5'-ACACATCTCGGCTCAATTTGC-3'; reverse: 5'-AGTGTCCACAACATGCTCCAT-3'. Primers for miR-101, U6, Cel-mir-39 and let-7i-5p were purchased from Ribobio (Guangzhou, China). The 2^-∆∆CT^ method was applied to determine relative miRNA and mRNA expression levels.

### CCK-8 assay

After lentiviral vector-mediated transduction, 3000 of the indicated osteosarcoma cells were seeded in each well of 96-well plates, with 6 wells per group, and the CCK-8 assay was performed at 24, 48, 72 and 96 h with detection of the absorbance at 450 nm after incubation with 10 μl of CCK-8 reagent (MCE, USA) for 1 h.

### Western blotting analysis

Extracted protein was separated on 6% or 10% sodium dodecyl sulfate (SDS)-polyacrylamide gel electrophoresis (PAGE) gels and transferred onto polyvinylidene difluoride membranes (BD Biosciences). Antibodies to BCL6 (1:250 overnight incubation at 4 ℃; CST), TSG101, CD63, ALIX, HSP70 and APOB (1:1000 overnight incubation at 4 ℃; Proteintech) were used with the same secondary antibody horseradish peroxidase (HRP)-labeled goat-anti-rabbit (1:5000; 1 h incubation at room temperature; Proteintech), while β-actin (1:1000 overnight incubation at 4 ℃; Proteintech) was used with the secondary antibody HRP-labeled goat-anti-mouse (1:5000; 1 h incubation at room temperature; Proteintech). Blots were detected by chemiluminescence using the ChemiDoc XRS Imaging System (Bio-Rad).

### Dual-luciferase reporter assay

The wild-type and mutant 3'UTRs of BCL6 were inserted into the Xhol and Ntol restriction sites of psiCHECK2 vector (Promega). The cells were plated into 96-well plates for 24 h and then transfected with BCL6 wild-type or mutant 3'UTR vector using Lipofectamine 2000 reagent (Invitrogen). Cells were lysed 48 h after transfection, and luciferase activity was then determined by the Luc-Pair^TM^ Duo-Luciferase HS Assay Kit (GeneCopoeia). The primer for the 3'UTR of BCL6 was forward: 5'-ATCGCTCGAGACTTCACTTGCGCCAGAA-3'; reverse: 5'-ATATGCGGCCGCGTGGATGAAAGAGGCACTACA-3'.

### Transient transfection with BCL6 plasmid

After plating into 6-well plates for 24 h, cells were transfected with GV492-BCL6 plasmid overexpressing BCL6 (Genechem) using Lipofectamine 2000 reagent according to the manufacturer's directions.

### Isolation and identification of AD-MSCs

Subcutaneous adipose tissues were acquired from three male patients (21, 23 and 31 years old) undergoing fracture treatment surgery at the Tangdu Hospital in Xi'an. AD-MSCs were isolated following a previously described protocol 36 and cultured in DMEM/F12 media (Gibco) containing 10% FBS (Gibco), 5 µg/ml Cellmaxin plus and 1% fibroblast growth factor (ScienCell) at 37 °C with 5% CO_2_. The surface markers of AD-MSCs (passage 3) were detected by flow cytometric analysis (NovoCyte 2040R; ACEA, USA) by using phycoerythrin (PE)-labeled CD31, CD34, CD44, CD45, CD90 and CD105 (BD Biosciences). PE-labeled IgG1 was used as an isotype control. Multi-lineage differentiation of AD-MSCs into adipocytes, chondrocytes and osteocytes was evaluated using Oricell^®^ adipogenesis, chondrogenesis and osteogenesis differentiation kits (Cyagen Biosciences, China). Cells were used at passages 3-6.

### Isolation of EVs

For EV isolation, 1.2×10^6^ AD-MSCs (AD-MSCs-101 or AD-MSCs-NC) were seeded into a 75-cm^2^ flask (Corning) in DMEM/F12 media with 10% FBS. After 24 h, the medium was replaced with 8 ml fresh DMEM/F12 media with 10% exosome-free FBS (SBI). After incubation for 48 h, the media was collected and processed with differential centrifugation. The obtained particles were suspended in PBS, and the number of the particles was measured by NTA to be (5.8±0.31)×10^8^/flask from AD-MSCs-101 and (5.8±0.42)×10^8^/flask from AD-MSCs-NC. EVs were collected from the supernatant by differential centrifugation 37. Briefly, the collected supernatant underwent differential centrifugation at 300×g for 5 min and 3000×g for 20 min, prior to 6000×g for 40 min and 10000×g for 60 min. To obtain a pellet of EVs, the supernatant was then ultracentrifuged at 100,000×g for 60 min, resuspended in PBS and again ultracentrifuged at 100,000×g for 60 min.

Whole blood was collected in an EDTA anticoagulant-containing tube, and plasma was obtained by centrifugation at 3000 rpm for 10 min at 4 ℃. Aliquots of plasma were immediately stored at -80 ℃ for further use. For later isolation of EVs, 1.5 ml of human plasma was diluted in PBS to 10 ml and then separated by differential centrifugation according to the method described above.

### Transmission electron microscopy

Isolated EVs were resuspended in PBS and transferred onto a carbon-coated copper grid. After air drying, EVs were stained with phosphotungstic acid. Images were captured after drying by TEM (JEM-1230; JEOL, Japan).

### Evaluation of EV uptake

The PKH67 Green Fluorescent Cell Linker Kit (Sigma) was used to label EVs. EVs were obtained from the collected supernatant by differential centrifugation as described above. First, 0.5 ml Diluent C was used to resuspend the EVs, and then 4 μl PKH67 was added to the EV suspension. After incubation at room temperature for 4 min, 10 ml DMEM/F12 media containing 10% FBS was added to terminate the dyeing. Differential centrifugation was performed again to obtain a pellet of EVs. The obtained EVs were added into the culture media of osteosarcoma cells. After incubation with PKH-67-labeled EVs for 8 h, cells were fixed with 4% paraformaldehyde for 10 min and then stained with phalloidin (cytoskeletal stain) for 30 min and DAPI (4′,6-diamidino-2-phenylindole) for 5 min (Sigma-Aldrich), separately. Finally, fluorescence images were captured using a FV1000 laser scanning confocal microscope (Olympus).

### Nanoparticle tracking analysis

ZetaView (Particle Metrix, Germany) was used for the characterization of vesicles. Each sample was diluted to 3 ml in PBS, and then the particle size distribution and concentration were analyzed three times for each sample.

### Migration and invasion assays

Osteosarcoma cells (1×10^5^ /well) were seeded in each well of 6-well plates. After 24 h, the media was replaced with fresh media. EVs (obtained from six 75-cm^2^ flasks of conditioned media of AD-MSCs-NC or AD-MSCs-101) or an equal volume of PBS was added to the culture media. After incubation for 48 h, 8×10^4^ cells were added to the upper Transwell chambers (24-well insert; pore size, 8 μm; Corning, USA) precoated without (migration assay) or with (invasion assay) 50 μl of diluted Matrigel (1:8 dilution; BD Biosciences). Medium containing 10% FBS was added to the lower Transwell chambers. For the 143B cells, the transmigrated cells were fixed in 95% ethanol and stained with 0.5% crystal violet after culture for 12 or 20 h. For the other osteosarcoma cell lines, the culture time was 20 or 30 h.

### Wound healing assay

Osteosarcoma cells (2×10^5^/well) were seeded in each well of 6-well plates. After 24 h, the same number of EVs as used in the Transwell assay was added to the fresh culture media. After incubation with EVs for 48 h, the cell monolayer was scratched with a 1-ml pipette tip. Cells were then cultured in serum-free medium, and the migration ability was assessed at 0 h and 48 h under brightfield microscopy.

### Lung metastasis model and AD-MSC-derived EV-mediated therapy *in vivo*

All animal experiments complied with ethical regulations and were approved by the Medical Ethics Committee of the Fourth Military Medical University. For the lung metastasis model, 3×10^6^ (in 100 μl PBS) of the indicated cells were injected through tail vein of male Balb/c nude mice (6 weeks old). After 7 weeks of tumor growth, all mice were sacrificed, and the number of metastatic lung nodules and lung weight were assessed.

For the AD-MSC-derived EV therapy, 143B-luciferase cells (2×10^6^ cells in 20 μl PBS) were injected into the intramedullary cavity of tibia of male Balb/c nude mice (6 weeks old) for the development of lung metastasis 38, 39. At 14 days after injection, mice were divided into three groups according to equivalent ranges of tumor sizes (n=6 per group). The mice received repeat doses of 2.32×10^9^ EVs or an equal volume of PBS via tail vein injection two times a week for 1 month. After 7 weeks of tumor growth, an IVIS was used to monitor tumor development. Then, all mice were sacrificed, and the number of metastatic lung nodules and lung weight were assessed.

For the evaluation of the *in vivo* safety of EV treatment, Balb/c nude mice (6 weeks old) were divided into three groups (n=5 per group) and given the same doses of EVs or an equal volume of PBS via tail vein injection two times a week for 1 month. One week after the last injection treatment, all mice were sacrificed. The levels of serological markers were tested, and H&E staining of major organs was performed to assess organ toxicity.

### Immunohistochemistry

The miR-101 detection probe for ISH was synthesized by Servicebio (Wuhan, China). The miR-101 probe sequence was 5'-TTCAGTTATCACAGTACTGTA-3'. Anti-digoxigenin-HRP (anti-DIG-HRP) antibody (Jackson Laboratories, USA) and DAB (Servicebio) were used for detection. Immunoreactivity scores were assigned based on the intensity and proportion of positive cells as described previously 37 and defined as follows: 0-4, low expression; 5-12, high expression.

### Next-generation sequencing

Plasma EVs were isolated from one osteosarcoma patient (17 years old, female) and one healthy control (17 years old, female) using Ribo^TM^ exosome isolation reagent (Ribobio). cDNA libraries were constructed with the EV-RNA, and then the sequencing was performed on the Illumina HiSeqTM 2500 platform (Illumina, USA). Clean reads were acquired after qualification of raw reads using FastQC software (www.bioinformatics.babraham.ac.uk/projects/fastqc/) and then mapped to a reference genome by Burrows-Wheeler Aligner software (http://bio-bwa.sourceforge.net/). Then, the obtained sequences were aligned against the miRNA database miRbase version 21 (www.miRBase.org), and known miRNAs were verified using miRDeep2 software (http://www.mdc-berlin.de/rajewsky/miRDeep). The miRNA expression values were normalized by the following RPM (reads per million) formula: RPM = (number of reads mapping to miRNA/number of reads in clean data) × 10^6^. The significantly differentially expressed miRNAs were determined according to the criteria of |log2 (fold change) |≥1 and a *P*-value <0.05.

### Statistical analysis

Statistical tests were performed using GraphPad Prism 7 software (GraphPad, USA) and SPSS 22.0 software (IBM, USA). Unless otherwise noted, data are shown as the mean ± standard error of mean (SEM). Statistical significance was assumed at *P*<0.05.

## Supplementary Material

Supplementary figures and tables.

## Figures and Tables

**Table 1:** Clinical characteristics of osteosarcoma patients stratified by low and high expression of miR-101.

Characteristics	n	miR-101 expression		*P*
		Low, n	High, n.	
Age (years)				
≤18	72	33	39	0.6673^a^
>18	42	21	21	
Gender				
Male	69	35	34	0.3742^a^
Female	45	19	26	
Tumor size (cm^2^)				
≤8	44	17	27	0.1388^a^
>8	70	37	33	
Clinical stage				
IB-IIA	24	6	18	0.0014^b^
IIB/III	90	48	42	
Distant metastasis				
Yes	48	30	18	0.0058^a^
No	66	24	42	

*^a^P*-values were calculated using Chi-square test. ^b^*P*-value was calculated using Mann-Whitney test.

**Table 2:** Screening of plasma EV-miRNAs in an osteosarcoma patient compared to a healthy control using NGS (partial results).

miRNAs	Osteosarcoma (mean reads)	Healthy control (mean reads)	Log2 (fold change)	*P*
miR-142-5p	531.98	3584.16	-2.7522	1.97E-06
miR-491-5p	39.73	175.74	-2.1451	1.26 E-04
miR-495-3p	47.48	5.91	3.0061	5.989E-03
miR-21-3p	22.29	55.63	-1.3195	8.679E-03
**miR-101-3p**	**934.12**	**2258.99**	**-1.274**	**4.123E-03**
**let-7i-5p**	**60115.27**	**67706.78**	**-0.1716**	**0.200366**

## Abbreviations

AD-MSCs: adipose tissue-derived mesenchymal stromal cells
AD-MSC-miR-101-EVs: AD-MSC-101-derived EVs
AD-MSC-NC-EVs: AD-MSC-NC-derived EVs
anti-DIG-HRP: anti-digoxigenin-horseradish peroxidase
BCL6: B cell lymphoma 6
EV: extracellular vesicle
EV-miRNAs: EV-packaged miRNAs
EV-miR-101: EV-packaged miR-101
EV-let-7i-5p: EV-packaged let-7i-5p
EZH2: enhancer of zeste homolog 2
GFP: green fluorescent protein
H&E: hematoxylin and eosin
ISH: in situ hybridization
IVIS: *in vivo* imaging system
miRNA: microRNA
MOI: multiplicity of infection
NTA: nanoparticle tracking analysis
NGS: next-generation sequencing
ROC: receiver operating characteristic
RPM: reads per million
TEM: transmission electron microscopy
3'UTR: 3' untranslated region
