# Supplementary material for: Extracellular vesicle-mediated delivery of miR-101 inhibits lung metastasis in osteosarcoma
Source: Theranostics. 2020 Jan 1;10(1):411–25. doi: 10.7150/thno.33482 (PMC6929625; doi:10.7150/thno.33482)
Supplement: Supplementary file 1 — Supplementary figures and tables. [file thnov10p0411s1.pdf]

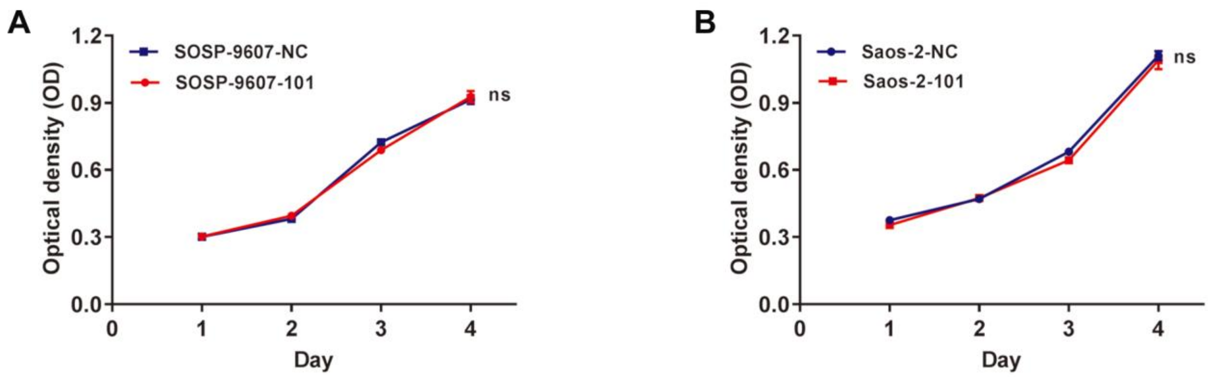


Figure S1. Effect of miR-101 on the proliferation of osteosarcoma cells observed via CCK-8 assay. (A) CCK-8 assay was performed on SOSP-9607-101 and SOSP-9607-NC cells. (B) CCK-8 assay was performed on Saos-2-101 and Saos-2-NC cells. Two-way ANOVA and Tukey’s multiple comparisons test. ns, not significant ($P$ >0.05).

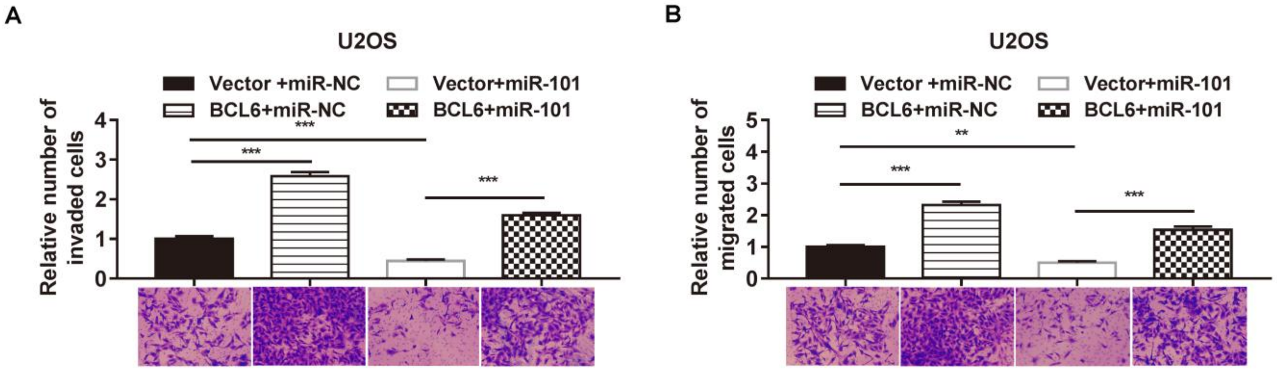


Figure S2. Effect of restoration of BCL6 expression on U2OS cell invasion and migration observed via Transwell assay. (A) Transwell invasion assay. (B) Transwell migration assay. One-way ANOVA and Tukey's multiple comparisons test. ** $P$ <0.01; *** $P$ <0.001.

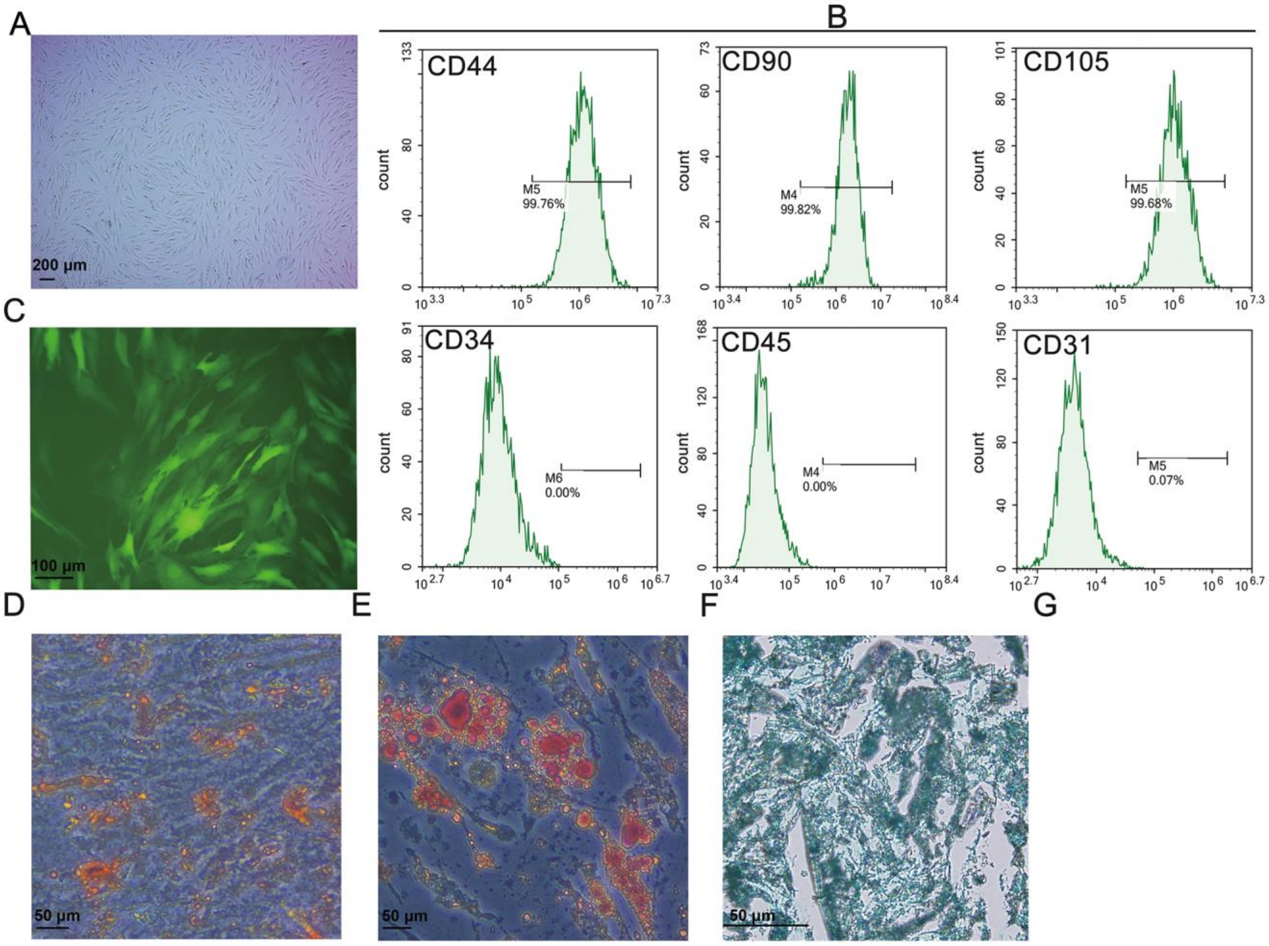


Figure S3. Identification of AD-MSCs and confirmation of the multi-lineage differentiation potential of engineered AD-MSCs. (A) Cellular morphology of AD-MSCs (passage 3) observed under light microscopy (40×). (B) Flow cytometric analysis of AD-MSC surface marker expression. (C) Successful lentiviral vector-mediated transduction of AD-MSCs confirmed by observation of GFP expression under fluorescence microscopy (100×). (D) Alizarin red S staining of engineered AD-MSCs after the induction of osteocyte differentiation (400×). (E) Oil red O staining of engineered AD-MSCs after the induction of adipocyte differentiation (400×). (F) Alcian blue staining of engineered AD-MSCs after the induction of chondrocyte differentiation (400×).

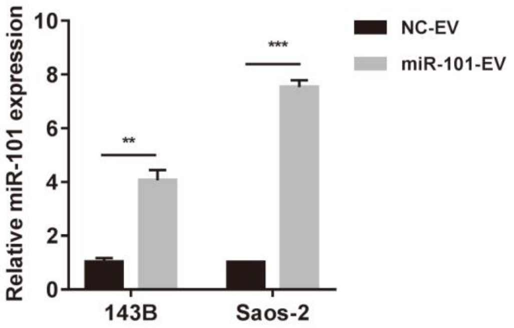


Figure S4. Detection of miR-101 expression in osteosarcoma cells, after uptake of AD-MSC-101-EVs by 143B and Saos-2 cells. NC-EV, EVs derived from AD-MSC-NC cells; miR-101-EV, EVs derived from AD-MSC-101 cells. Student's *t*-test. $^{**}$ $P$ <0.01; $^{***}$ $P$ <0.001.

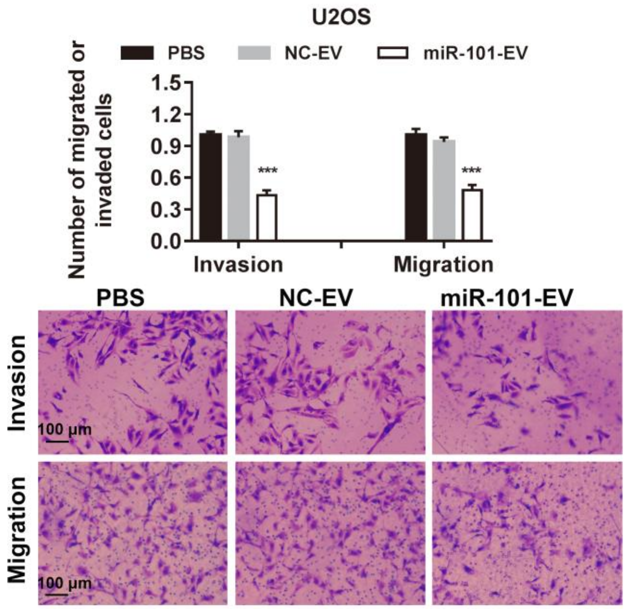


Figure S5. Effect of AD-MSC-101-EVs on invasion and migration abilities of U2OS cells observed via Transwell assay. NC-EV, EVs derived from AD-MSC-NC cells; miR-101-EV, EVs derived from AD-MSC-101 cells. One-way ANOVA and Tukey's multiple comparisons test. $^{***}$ $P$ <0.001 in comparison with PBS or NC-EV.

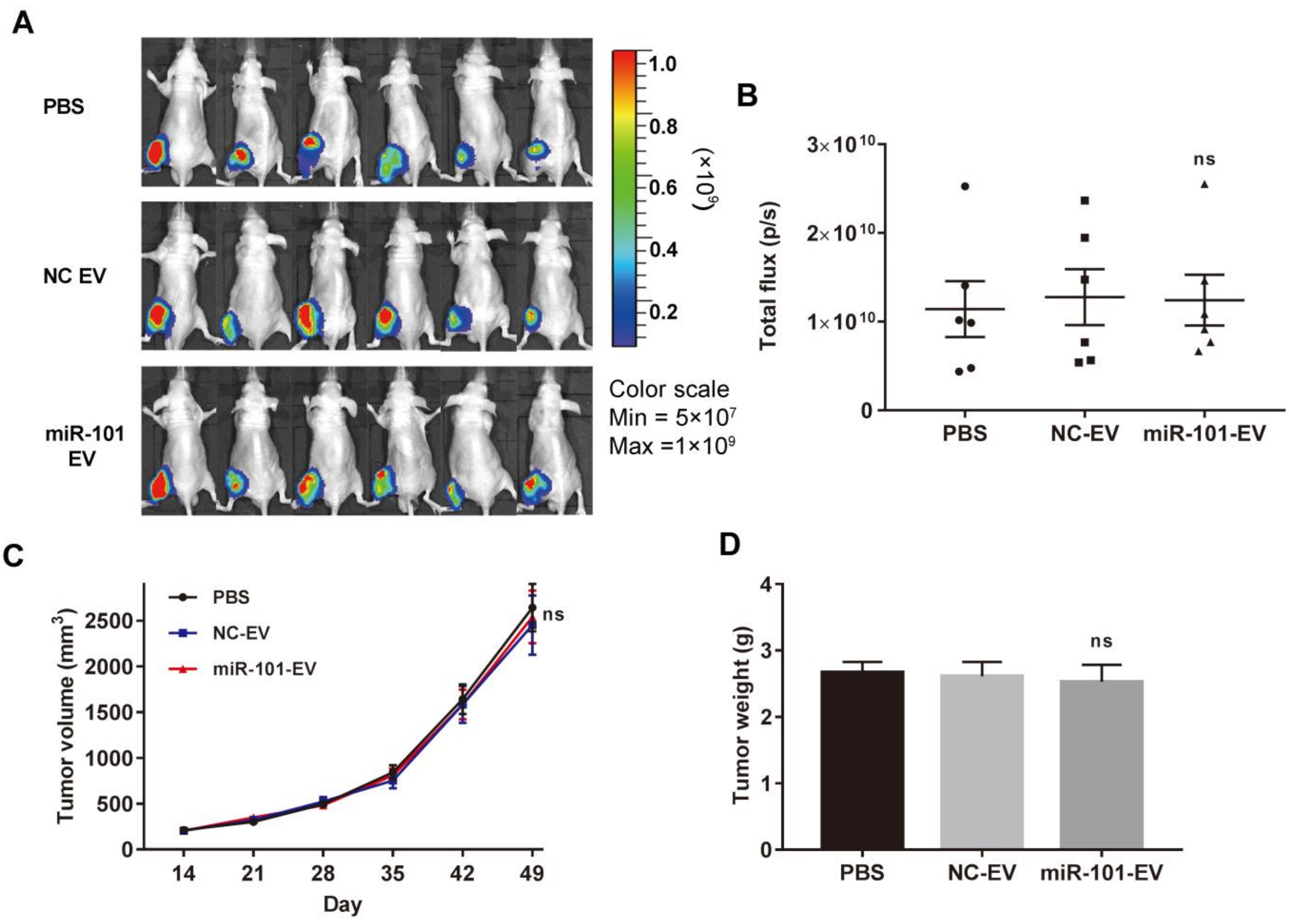


Figure S6. AD-MSC-101-EVs had little effect on the proliferation of osteosarcoma cells in vivo. NC-EV, EVs derived from AD-MSC-NC cells; miR-101-EV, EVs derived from AD-MSC-101 cells. (A) Primary tumors in the three groups were evaluated by IVIS at 7 weeks after tumor cell injection (n=6 per group). (B) Quantification of bioluminescence imaging. p/s: photons per second. Kruskal-Wallis test and Dunn's multiple comparisons test. (C) Proliferation curve for primary tumors. Volume = ½ × Length × Width$^2$. Two-way ANOVA and Tukey's multiple comparisons test. (D) Illustration of lung weight from the nude mice. One-way ANOVA and Tukey's multiple comparisons test. ns, not significant (*P* >0.05) in comparison with PBS or NC-EV.

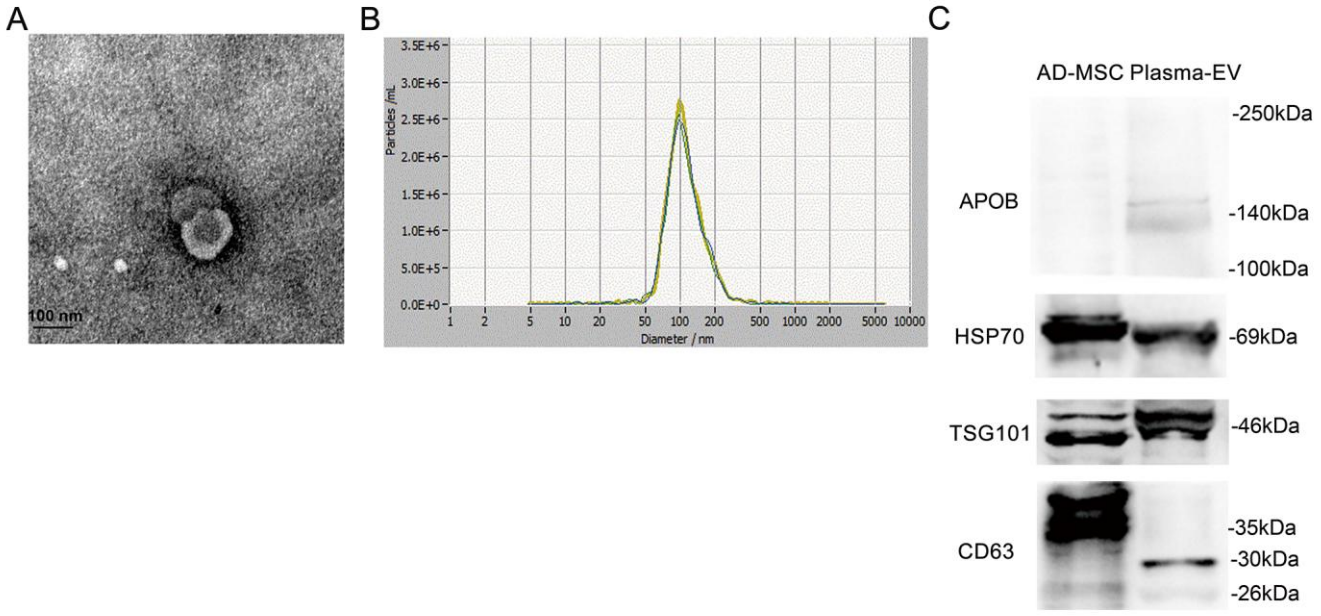


Figure S7. Characterization of plasma EVs. AD-MSC, adipose tissue-derived mesenchymal stromal cells; Plasma-EV, EVs derived from plasma. (A) Representative TEM of plasma EVs. (B) Results of NTA for plasma EVs (n=3). (C) Detection of surface markers of EVs was performed by western blotting. Briefly, 2 μg protein samples were loaded in each lane, and AD-MSC lysate was used as a control for EV characterization.

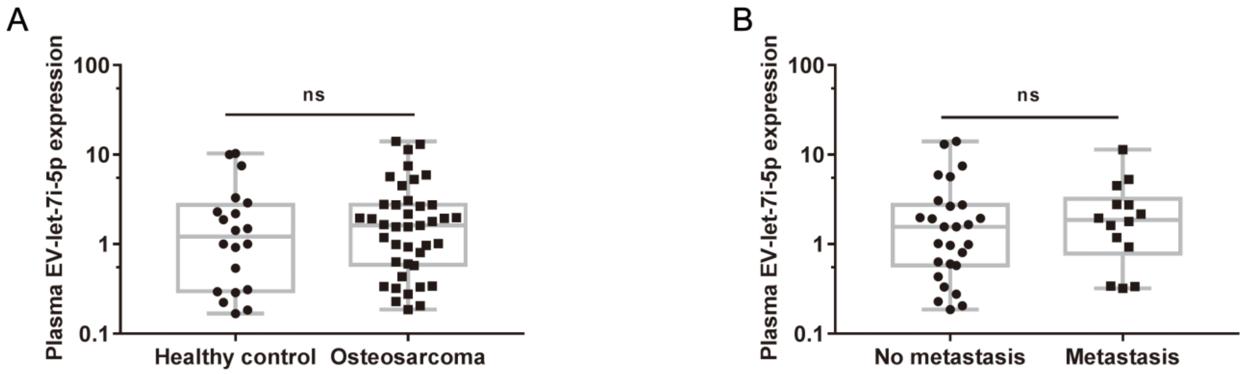


Figure S8. Plasma EV-let-7i-5p level was detected in healthy controls and osteosarcoma patients. (A) No significant difference in EV-let-7i-5p expression was observed between osteosarcoma patients and healthy controls. Mann-Whitney test. Error bars show minimum to maximum values. (B) No significant difference in EV-let-7i-5p expression was observed between osteosarcoma patients with metastasis and those without metastasis. Mann-Whitney test. Error bars show minimum to maximum values. ns, not significant ($P > 0.05$).

Table S1. Characteristics of healthy controls and osteosarcoma patients.

| Characteristics | Healthy controls (n=20) | OS patients (n=41) |
|---|---|---|
| Gender | | |
| Male | 12 | 27 |
| Female | 8 | 14 |
| Age (years) | | |
| Median | 18.5 | 16 |
| Range | 11-51 | 8-49 |
| Anatomical site | | |
| Femur | | 26 |
| Tibia | | 8 |
| Radius | | 3 |
| Pelvis | | 2 |
| Other | | 2 |
| Clinical stage | | |
| IIA | | 8 |
| IIB/III | | 33 |

OS: osteosarcoma
